# Supplementary material for: Legless soft robots capable of rapid, continuous, and steered jumping
Source: Nat Commun. 2021 Dec 7;12:7028. doi: 10.1038/s41467-021-27265-w (PMC8651723; doi:10.1038/s41467-021-27265-w)
Supplement: Supplementary file 1 — Supplementary Information [file 41467_2021_27265_MOESM1_ESM.pdf]

Supplementary Information

Legless soft robots capable of rapid, continuous, and steered jumping

Rui Chen[1†*], Zean Yuan[1†], Jianglong Guo[2†], Long Bai[1†], Xinyu Zhu[1], Fuqiang Liu[3], Huayan Pu[4*], Liming Xin[5], Yan Peng[6], Jun Luo[1,4], Li Wen[7], Yu Sun[8]

[1] State Key Laboratory of Mechanical Transmissions, Chongqing University, Chongqing 400044, China.

[2] School of Science, Harbin Institute of Technology (Shenzhen), Shenzhen 518055, China.

[3] College of Mechanical and Vehicle Engineering, Chongqing University, Chongqing 400044, China.

[4] School of Mechatronics Engineering and Automation, Shanghai University, Shanghai 200444, China.

[5] School of Computer Engineering and Science, Shanghai University, Shanghai 200444, China.

[6] Research Institute of Unmanned Surface Vessel Engineering, Shanghai University, Shanghai 200444, China.

[7] School of Mechanical Engineering and Automation, Beihang University, Beijing 100191, China.

[8] Department of Mechanical and Industrial Engineering, University of Toronto, Toronto, Canada.

[†] These authors contributed equally to this work.

[*] Correspondence and requests for materials should be addressed to R.C. (email: cr@cqu.edu.cn) or to H.P. (email: phygood_2001@shu.edu.cn).

Supplementary Note 1. Qualitative analysis of the locomotion mechanism

Under squeezing by the electrodes after the application of a voltage, a portion of the dielectric liquid flows anisotropically, resulting in a horizontal initial kinetic energy. This part of the kinetic energy is eventually converted into the kinetic energy of the entire actuator to provide energy for forward jumping. As shown in Supplementary Fig. 6, we assumed that the area ratio of the electrode area (semicircle area) to the nonelectrode area (semiring area) was 1:1, and after applying a voltage, all the liquid in the electrode area would flow into the nonelectrode area. Therefore, according to the calculation formula of the center of gravity of semicircle and semiring, the moving distance of the center of gravity (red dots) of this part of the dielectric liquid can be calculated as:

$$\Delta x = \frac{4}{3\pi} \cdot \frac{R_1^{\,2} + R_2^{\,2} + R_1 \cdot R_2}{R_1 + R_2} - \frac{4}{3\pi} \cdot R_2 \tag{S1}$$

where R_1 and R_2 are the radius of the dielectric liquid pouch and the radius of the electrode area, respectively. Here, we selected R_1 = 27.5 mm as the radius of the dielectric liquid pouch and R_2 = 19.5 mm as the radius of the electrode area to ensure that the area ratio of the electrode area to the nonelectrode area was 1:1. The value of Δx was calculated to be approximately 6.829 mm.

The horizontal flow speed of the moving dielectric liquid in the electrode area can be calculated as follows:

$$v_{\text{oil}} = \frac{\Delta x}{t} \tag{S2}$$

where Δx and t are the moving distance of the center of gravity of this partial dielectric liquid and the time it takes for the liquid to flow, respectively. Here, the liquid flow time is defined to be the same as the actuator deformation time, which is approximately 10 ms. The v_{oil} was calculated to be approximately 0.683 m/s.

The mass of the moving dielectric liquid in the electrode area can be calculated as:

$$\Delta m_{\text{oil}} = \Delta V_{\text{oil}} \cdot \rho \tag{S3}$$

where ΔV_{oil} and ρ are the volume of the moving dielectric liquid and the density of the dielectric liquid, respectively. The area ratio of the electrode area (semicircle area) to the nonelectrode area (semiring area) was 1:1, and after applying a voltage, all the liquid in the electrode area would flow into the nonelectrode area. Therefore, here, the volume of the moving dielectric liquid ΔV_{oil} is 0.5 mL, which is half of the dielectric liquid in the front pouch. The density of the 25# mineral transformer oil ρ = 895 kg/m^3. Δm_{oil} was calculated to be 0.4475 g.

The horizontal initial kinetic energy of the moving dielectric liquid in the electrode area can be calculated as:

$$E_{\text{k(oil)}} = \frac{1}{2} \Delta m_{\text{oil}} v_{\text{oil}}^{2} \tag{S4}$$

where Δm_{oil} and v_{oil} are the mass of the moving dielectric liquid in the electrode area and the horizontal flow speed of the moving dielectric liquid in the electrode area, respectively. The $E_{\text{k(oil)}}$ was calculated to be approximately 1.044×10^{-4} J.

We assume that this part of the kinetic energy would eventually be converted into the kinetic energy of the entire actuator to provide partial energy for forward jumping. Therefore, the horizontal jumping velocity provided to the liquid-air actuator (LSJR without frame) by the squeezed liquid anisotropic flow can be calculated as:

$$v_{x1} = \sqrt{\frac{2E_{\text{k(oil)}}}{m_{\text{LSJR}} - m_{\text{frame}}}} \tag{S5}$$

where $E_{\text{k(oil)}}$, m_{LSJR}, and m_{frame} are the horizontal initial kinetic energy of the moving dielectric liquid in the electrode area, the mass of the LSJR, and the mass of the PVC frame, respectively. Here, the m_{LSJR} was 1.10 g and the m_{frame} was 0.17 g. v_{x1} was calculated to be approximately 0.474 m/s.

Similarly, the horizontal jumping velocity provided to the liquid-air actuator with a plane frame (LSJR without prebending) and the liquid-air actuator with a prebending frame (LSJR) by the squeezed liquid anisotropic flow can also be calculated as:

$$v_{x2} = \sqrt{\frac{2E_{\text{k(oil)}}}{m_{\text{LSJR}}}} \tag{S6}$$

where $E_{\text{k(oil)}}$ and m_{LSJR} are the horizontal initial kinetic energy of the moving dielectric liquid in the electrode area and the mass of the LSJR, respectively. The v_{x2} was calculated to be approximately 0.436 m/s.

In Supplementary Fig. 7, the red line shows the theoretical values of the horizontal forward velocity provided to the actuators by the squeezed liquid anisotropic flow. Simultaneously, we tested the horizontal forward speeds of the LSJR without a frame, the LSJR without prebending, and the LSJR. The blue line shows these experimental values of the horizontal forward velocity and error

bars in Supplementary Fig. 7. One of the test results can be seen in Supplementary Fig. 8. As shown in Supplementary Fig. 7, the average horizontal forward speeds of the LSJR without a frame, the LSJR without prebending, and the LSJR are 0.404 m/s, 0.458 m/s, and 0.500 m/s, respectively. Their theoretical horizontal forward speeds provided by the squeezed liquid anisotropic flow are 0.474 m/s, 0.436 m/s, and 0.436 m/s, respectively. In the LSJR results, the measured horizontal forward speed was greater than the theoretical horizontal forward velocity provided to the actuator by the squeezed liquid anisotropic flow. LSJR's off-ground avoided the drag friction from the ground; and during the frame bending process, the ground surface provided additional forward friction to the front end of the LSJR and increased the forward speed. The prebending frame, which enabled the LSJR to take-off completely and converted the hindrance of friction into a positive power for forward jumping, played an important role in enhancing the jumping of electrohydraulic actuators.

Through experiments and analysis, one can see that the initial horizontal velocity of the LSJR is determined by the horizontal ground reaction forces at the frame ends, which are caused by the moving dielectric liquid flow and frame bending. Furthermore, the initial vertical velocity of the LSJR is determined by the vertical ground reaction forces at the frame ends, which are caused by the frame bending. Therefore, the ground reaction forces generated by the moving dielectric liquid flow and frame bending during the deformation process of the LSJR (Supplementary Fig. 9) need to be analyzed. Supplementary Fig. 9a shows snapshots of the LSJR during jumping, where 10 kV was applied to the actuator. A cross-sectional view of the robot is shown in Supplementary Fig. 9b, where the middle point of the ring frame was simplified to a joint, and the front part (from the joint to the front part) and the rear part (from the joint to the rear part) were simplified to two cantilevers. G denotes the force due to gravity. Assuming the robot is in the deformation process at this instance under a driving voltage, the directions of the ground reaction forces at the front end (F_f) and rear end (F_r) are shown in Supplementary Fig. 9b. According to the center of gravity and posture of LSJR after take-off, F_f is theoretically larger than F_r. The angle and size of F_f and F_r change with time t; thus, F_f and F_r can be assumed to be functions $F_f(t)$ and $F_r(t)$. Since the front end slips during the deformation process, the ground reaction force F_f was decomposed into the horizontal component sliding friction f and the vertical component F_{fy}, and the ground reaction force F_r was decomposed into the horizontal component static friction F_{rx} and the vertical component F_{ry}, and all the values change over time. The vertical impulse can be calculated as:

$$\int_0^t (F_{fy} + F_{ry})dt = m_{\mathrm{LSJR}} \cdot v_y \tag{S7}$$

where F_{fy} and F_{ry} are the vertical components of the ground reaction forces at the front end (F_f) and rear end (F_r); t is the time required for the deformation process; m_{LSJR} is the mass of the LSJR; v_y is the initial vertical velocity of aerial LSJR.

The horizontal impulse can be calculated as:

$$\int_0^t (F_{fy} \cdot \mu - F_{rx})dt = m_{\mathrm{LSJR}} \cdot v_x \tag{S8}$$

where F_{fy} and F_{rx} are the vertical component of the ground reaction force at the front end (F_f) and the horizontal component of the ground reaction force at the rear end (F_r); μ is the friction

coefficient between the BOPP film and the ground; t is the time required for the deformation process; m_{LSJR} is the mass of the LSJR; v_x is the initial horizontal velocity of aerial LSJR provided by the moving dielectric liquid flow and the bending of frame.

Therefore, the initial horizontal velocity and initial vertical velocity of aerial LSJR can be calculated as:

$$\begin{cases} v_x = \dfrac{1}{m_{\mathrm{LSJR}}} \displaystyle\int_0^t (F_{fy} \cdot \mu - F_{rx}) dt \\ v_y = \dfrac{1}{m_{\mathrm{LSJR}}} \displaystyle\int_0^t (F_{fy} + F_{ry}) dt \end{cases} \quad \text{(S9, S10)}$$

where F_{rx}, F_{fy}, and F_{ry} are the horizontal component of the ground reaction force at the rear end (F_r) and the vertical components of the ground reaction forces at the front end (F_f) and rear end (F_r), respectively; m_{LSJR} is the mass of the LSJR; μ is the friction coefficient between the BOPP film and the ground; t is the time required for the deformation process. Note that Supplementary Eq. 9 is only applicable when LSJR is subject to sliding friction at the front end and static friction at the rear end during the jumping process.

Here, F_{rx}, F_{fy}, and F_{ry} are determined by the moving dielectric liquid flow and the bending of frame. They are functions of deformation time t related to material (electrode, film, dielectric liquid, etc.), structure (prebending angle, electrode area/nonelectrode area ratio, size, etc.), and application voltage. It is challenging to calculate the exact values in this complex solid-liquid-gas system. Moreover, μ is related to the surface roughness of the substrate surface; m_{LSJR} is related to how much load is carried. Therefore, to optimize the jumping performance of LSJR, which is related to the initial horizontal velocity v_x and initial vertical velocity v_y of aerial LSJR, the following parameters can be varied and optimized: voltage, electrode area/nonelectrode area ratio, carried load, prebending level, and substrate surface roughness.

Supplementary Note 2. Precision test of continuous forward jumping and turning.

Under the applied voltage of 4 Hz and 10 kV, the single-unit LSJR can be used to conduct a 37.5 cm long translational motion by continuous forward jumping. Five trajectories of continuous jumps are shown in Supplementary Fig. 12a. When the robot reached the end, the lateral deviation averaged 0.62 cm (maximum 1.2 cm), and the angle deviation averaged 0.95° (maximum 1.67°). The whole jumping process can be seen in Supplementary Fig. 12b and Supplementary Movie 8.

Under the applied voltage of 4 Hz and 10 kV, the dual-body LSJR completed a 90° turn by continuously actuating one unit of the dual-body LSJR. Five trajectories of turns are shown in Supplementary Fig. 13a. When the robot turned 90° after five jumps, the angle deviation averaged 3.13° (maximum 5.13°). The whole turning process can be seen in Supplementary Fig. 13b and Supplementary Movie 8.

To move along a given route, the dual-body LSJR imitates the movement of bipedal animals, by alternately activating one of the two units in the robot. Continuous steering can adjust the positions to move on the predetermined route. The whole moving process can be seen in Supplementary Fig. 14 and Supplementary Movie 9. The dual-body LSJR can jump forward, turn or walk like two feet

to reach the given position and orientation. Smaller displacements can be achieved by lowering the applied voltage.

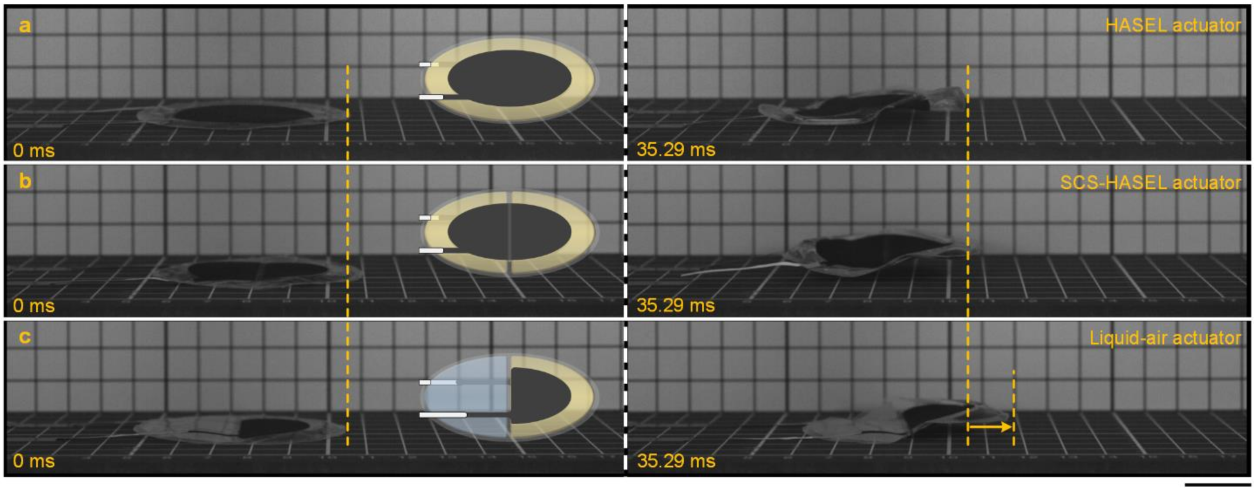

Supplementary Fig. 1. Special liquid-air layout and semicircular zipping structure. See also Supplementary Movie 1. (**a**) Snapshots of a HASEL actuator to which 10 kV was applied. (**b**) Snapshots of a SCS-HASEL actuator to which 10 kV was applied. (**c**) Snapshots of a liquid-air actuator to which 10 kV was applied. Scale bar, 2 cm.

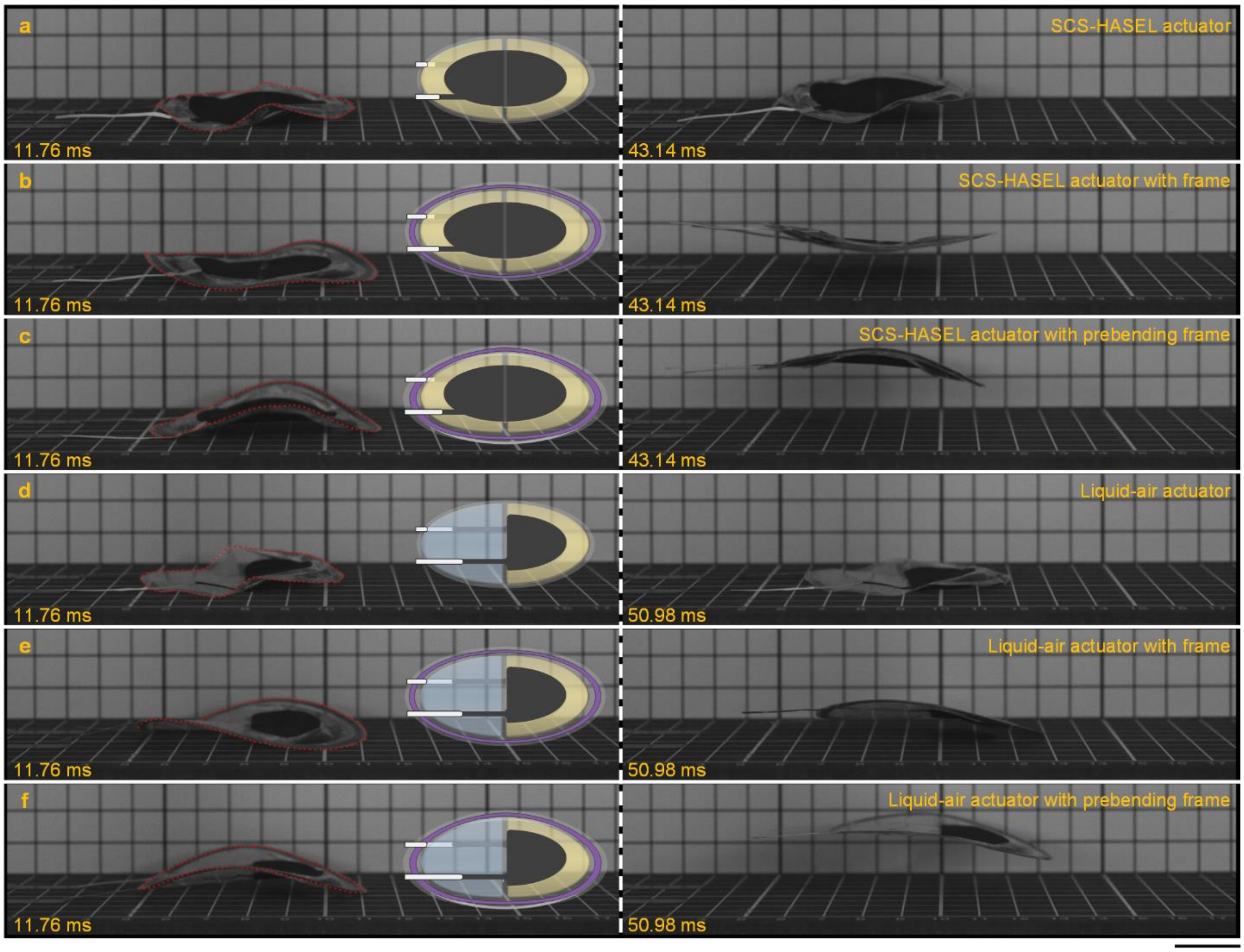

Supplementary Fig. 2. Jumping enhancement of frame and prebending. See also Supplementary Movie 1. (**a-c**) Comparison of the influence of frame and prebending on the jumping performance of a SCS-HASEL actuator. A voltage of 10 kV was applied to the actuators. (**d-f**) Comparison of the influence of frame and prebending on the jumping performance of a liquid-air actuator. A voltage of 10 kV was applied to the actuators. Scale bar, 2 cm.

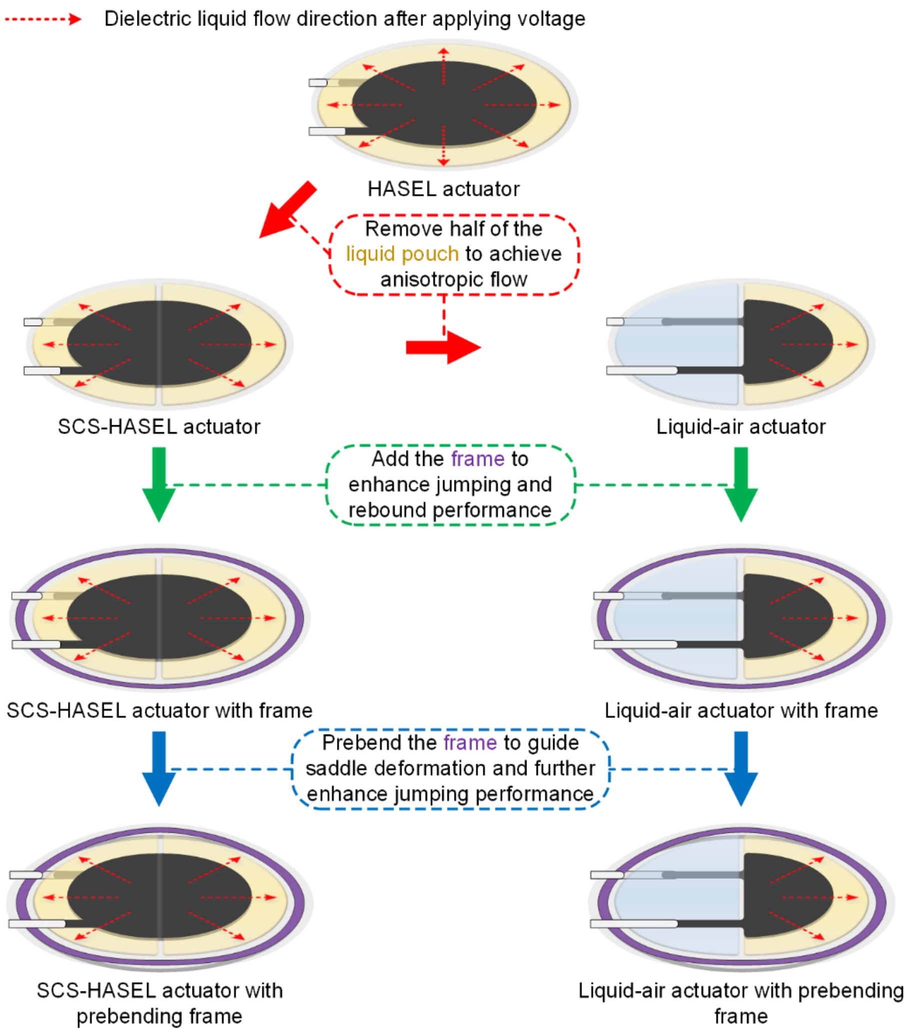

Supplementary Fig. 3. Design iterations of the LSJR.

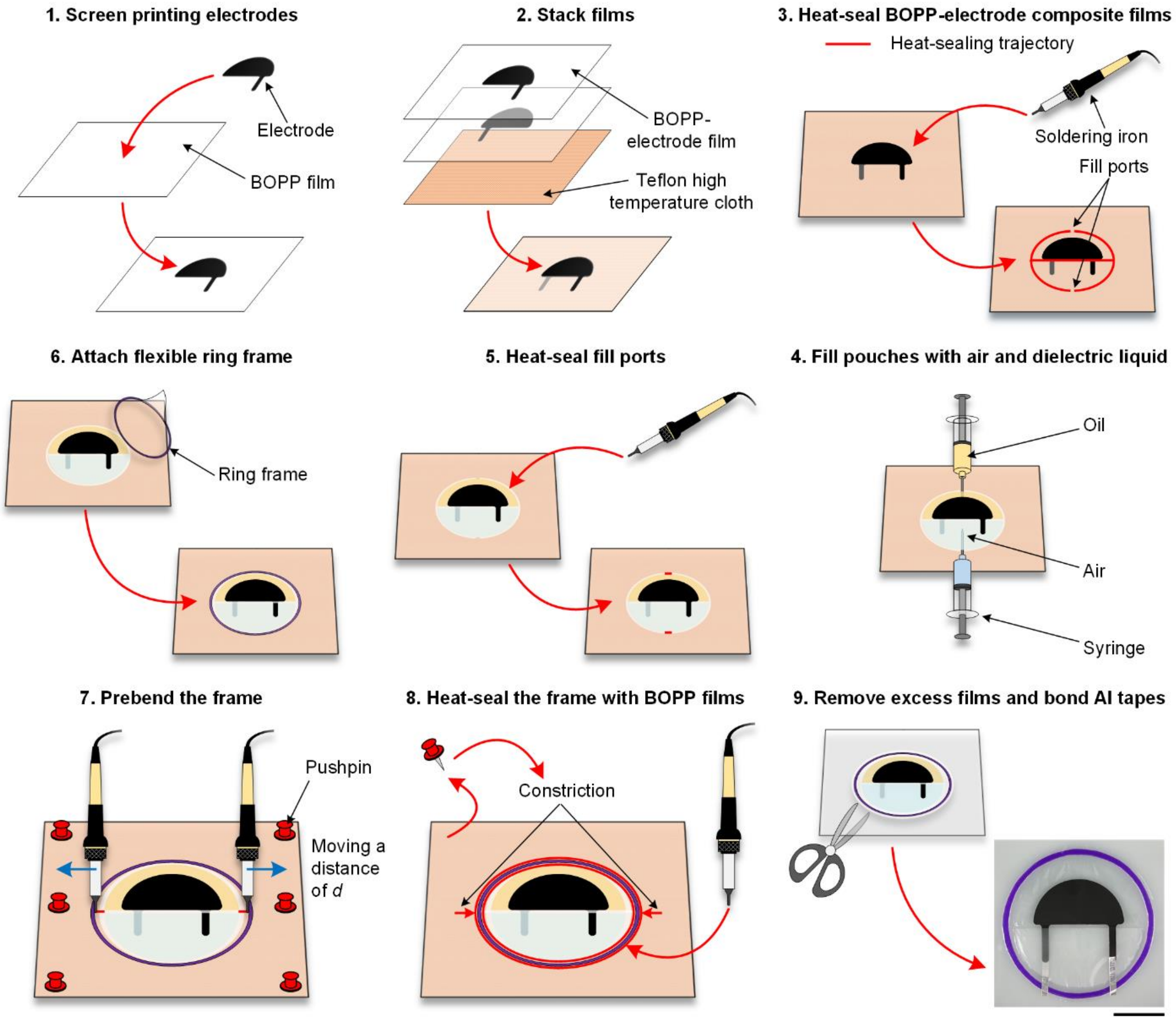

Supplementary Fig. 4. LSJR fabrication procedure. Scale bar, 2 cm.

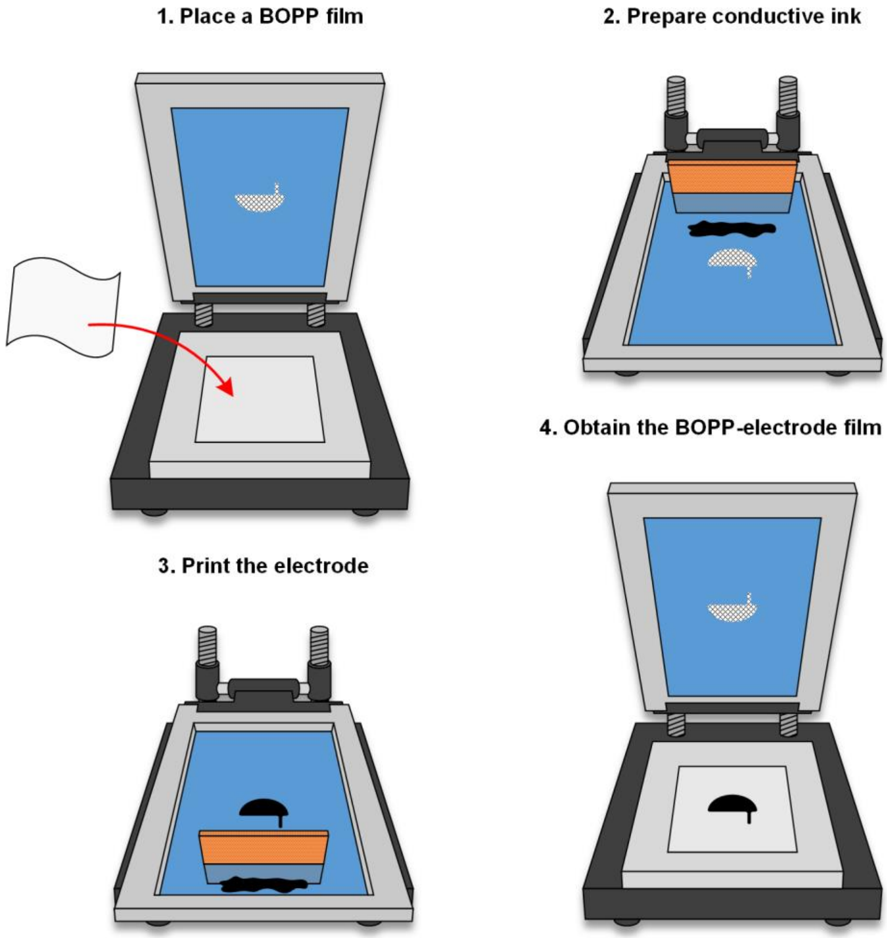

Supplementary Fig. 5. Electrode screen printing on BOPP films. First, we placed a BOPP film with the smooth surface facing up on the screen-printing machine. Second, we kept the BOPP film flat, covered the screen, and squeezed an appropriate amount of conductive ink onto the screen. Third, we used a squeegee to make the ink deposited through the screen onto the BOPP film. Finally, we lifted the screen off the substrate and obtained the BOPP-electrode film, which we cured at room temperature for 12 hours.

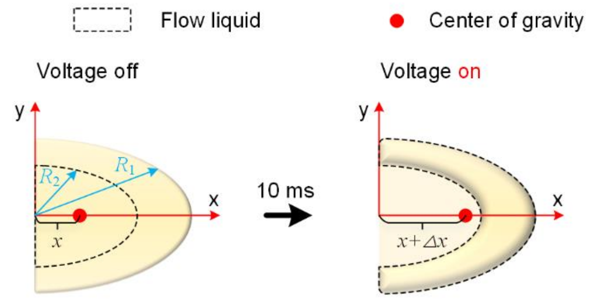

Supplementary Fig. 6. Dielectric liquid center of gravity movement equivalent model. The black dashed area and the red dot represent the flowing liquid and the center of gravity of the flowing liquid. The area ratio of the electrode area (semicircle area) to the nonelectrode area (semiring area) is 1:1. After applying a voltage for 10 ms, all the liquid in the electrode area flows into the nonelectrode area. The movement distance of the center of gravity is Δx.

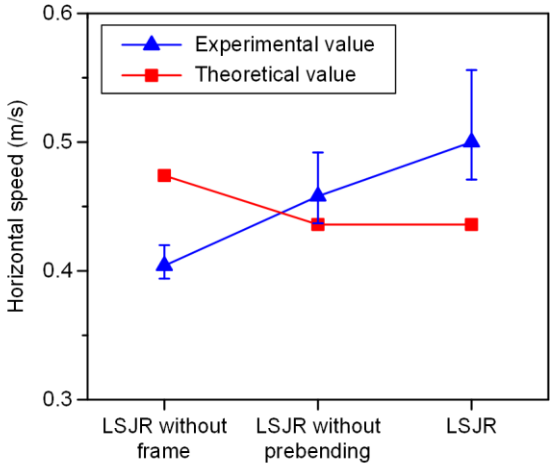

Supplementary Fig. 7. Actuators' horizontal speeds and the speeds provided by liquid flow. The red line represents the theoretical values of the horizontal forward velocity provided to the actuators by the squeezed liquid anisotropic flow. The theoretical values are 0.474 m/s, 0.436 m/s, and 0.436 m/s, respectively. The blue line represents the horizontal forward speeds of the LSJR without frame, the LSJR without prebending, and the LSJR. The average horizontal forward speeds are 0.404 m/s, 0.458 m/s, and 0.500 m/s, respectively.

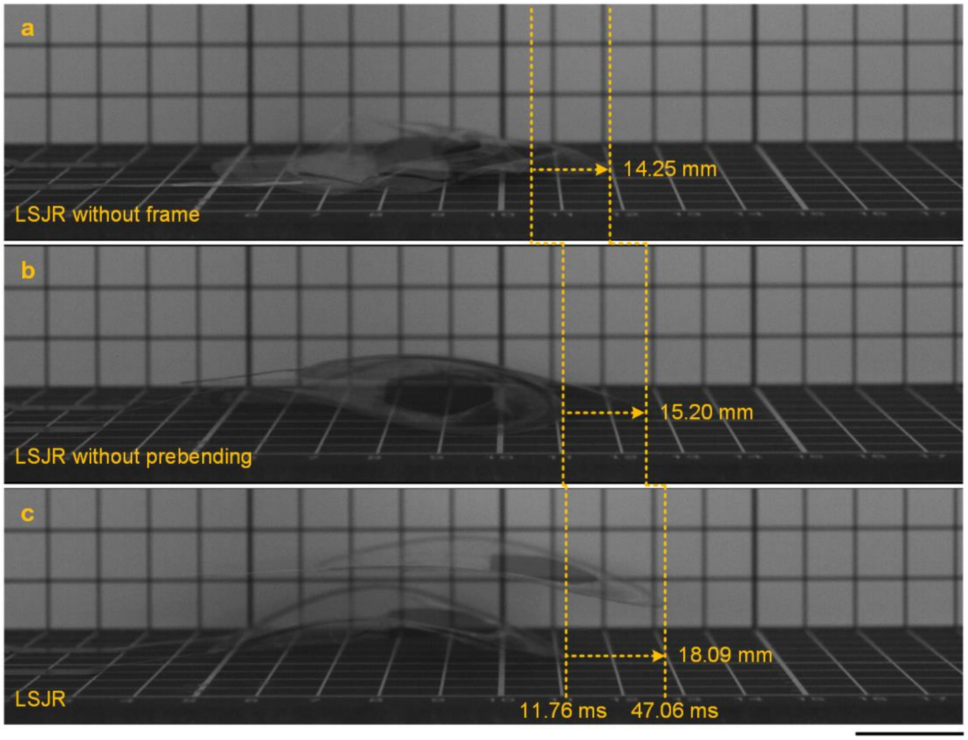

Supplementary Fig. 8. Composite image of the take-off posture and the aerial posture. See also Supplementary Movie 1. (**a**) The LSJR without frame and prebending. (**b**) The LSJR without prebending. (**c**) The LSJR. The yellow ladder dotted lines represent their forward displacements and time they spent. The horizontal forward speeds of these actuators can be calculated as 0.404 m/s, 0.431 m/s, and 0.512 m/s, respectively. As a simplification, the horizontal air resistance is ignored. Scale bar, 2 cm.

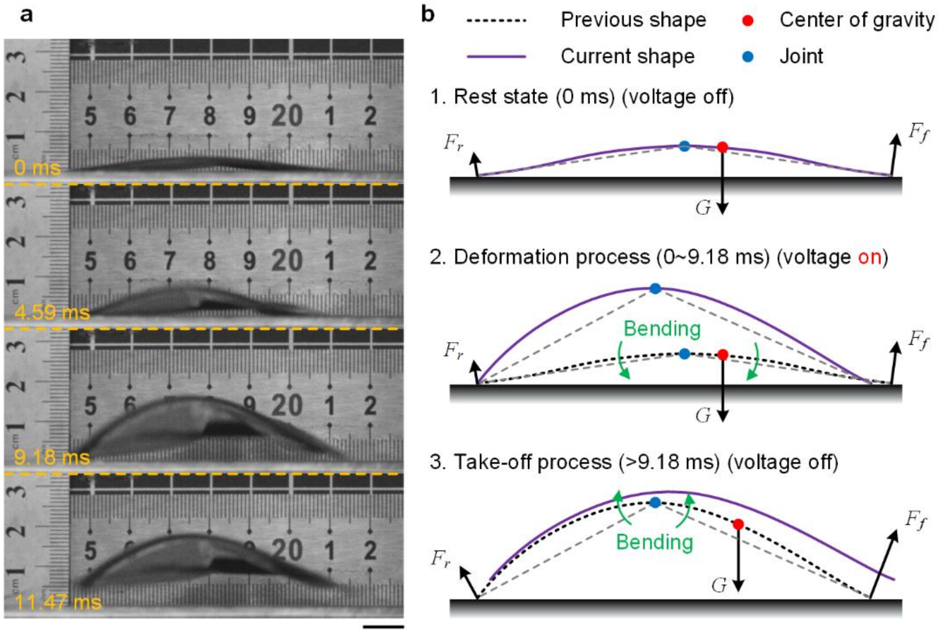

Supplementary Fig. 9. LSJR take-off process force analysis. See also Supplementary Movie 2. (**a**) Snapshots of the LSJR, where 10 kV was applied to the actuator. Scale bar, 1 cm. (**b**) The LSJR take-off process includes two main processes: the deformation process and the take-off process. The black dashed lines indicate the previous shapes, and purple solid lines are the current shapes.

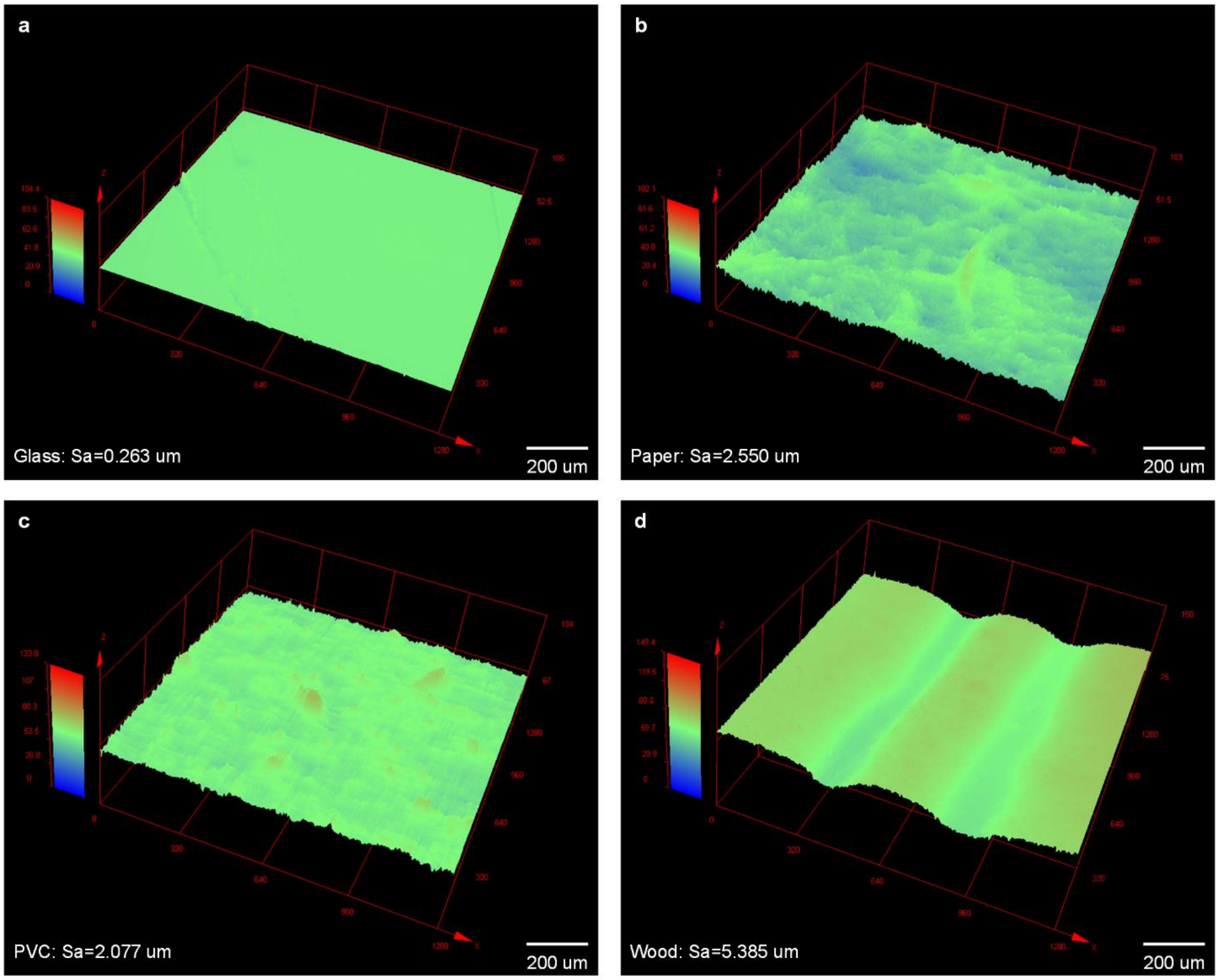

Supplementary Fig. 10. Surface textures of different substrates. (**a**) Glass, *Sa* = 0.263 µm. (**b**) Paper, *Sa* = 2.550 µm. (**c**) PVC, *Sa* = 2.077 µm. (**d**) Wood, *Sa* = 5.385 µm.

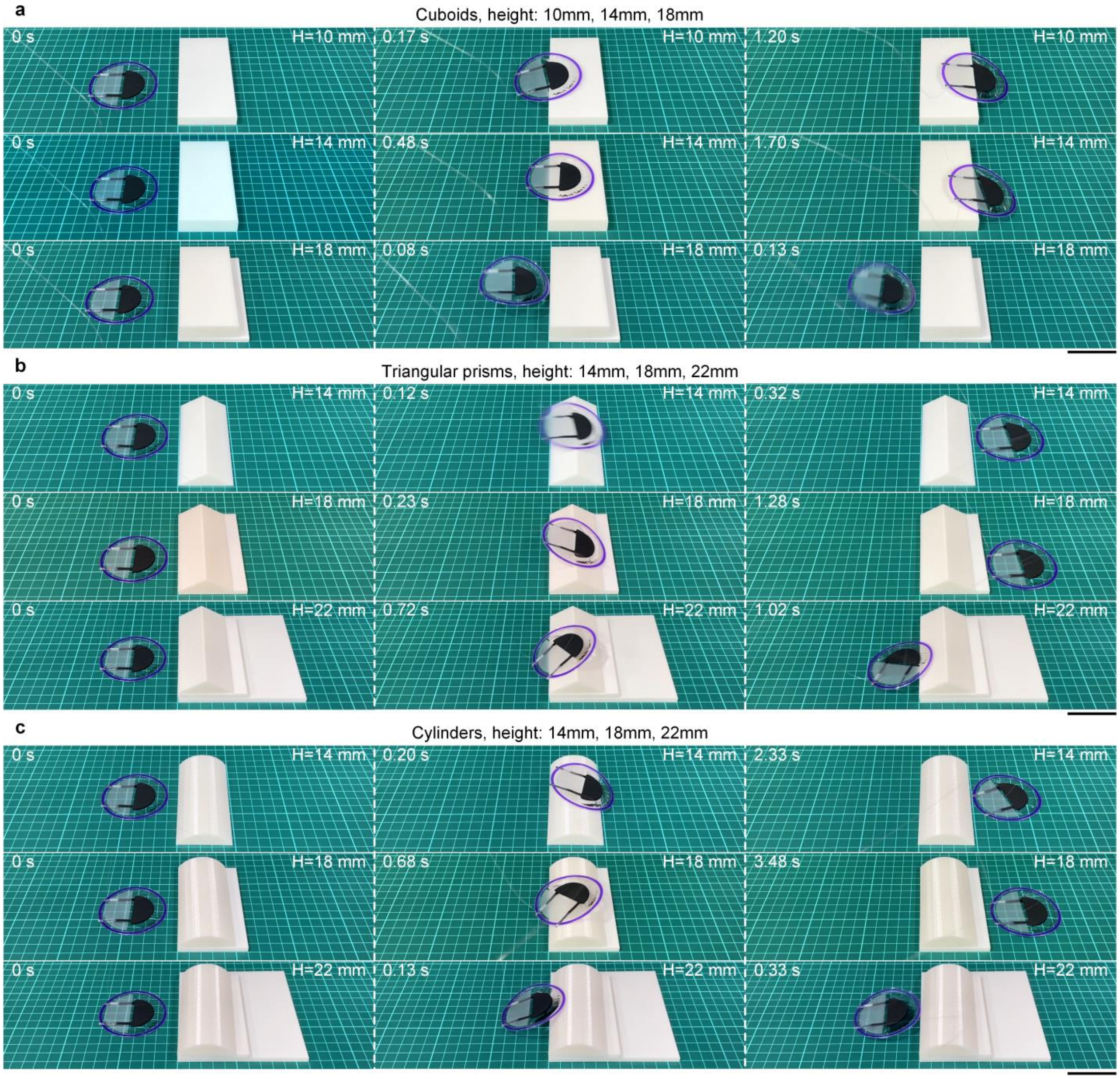

Supplementary Fig. 11. Single-unit LSJR crossing for obstacles of different shapes and sizes. See Supplementary Movie 7. (**a**) Crossing tests for three cuboids (height of 10 mm, 14mm and 18 mm). The LSJR collided with the front wall of the 18 mm height cuboid. (**b**) Crossing tests for three triangular prisms (height of 14 mm, 18mm and 22 mm). The LSJR slipped down from the 22 mm height triangular prism. (**c**) Crossing tests for three cylinders (height of 14 mm, 18mm and 22 mm). The LSJR slipped down from the 22 mm height cylinder. Scale bar, 4 cm.

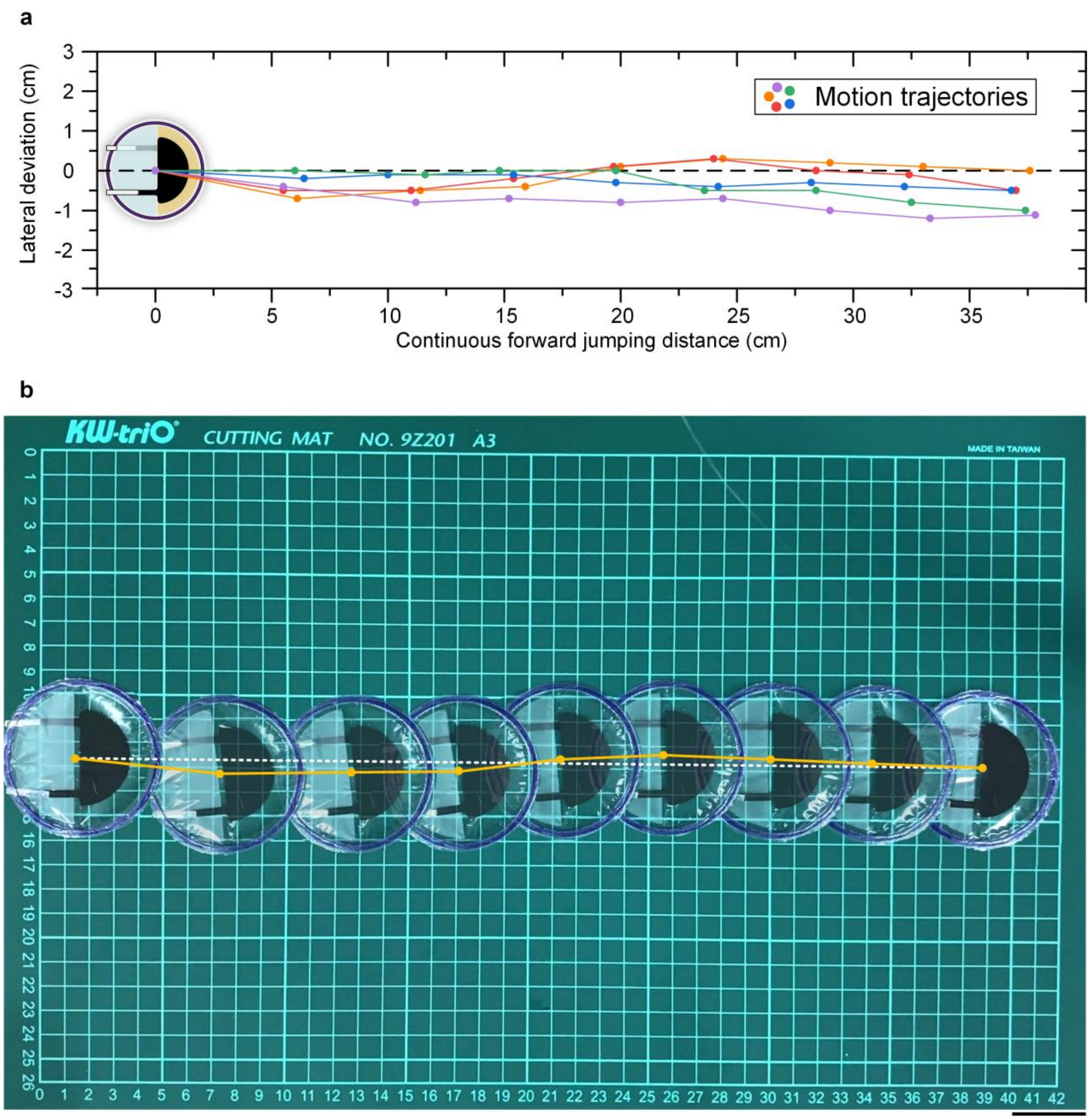

Supplementary Fig. 12. Continuous forward jumping trajectories of the single-unit LSJR. See Supplementary Movie 8. (**a**) Trajectories of five continuous forward jumps on the plate at 4 Hz and 10 kV. (**b**) Composite image of the initial position and seven landing points in continuous forward jumping of the yellow trajectory in the line chart. Scale bar, 4 cm.

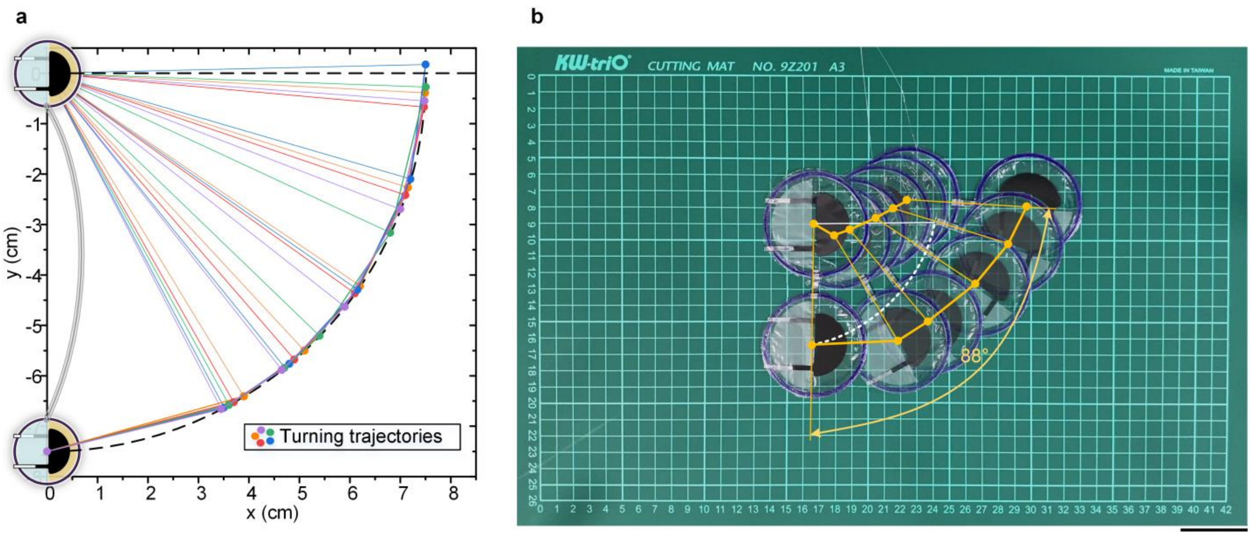

Supplementary Fig. 13. Turning trajectories of the dual-body LSJR. See Supplementary Movie 8. (**a**) Trajectories of five turning on the plate at 4 Hz and 10 kV. Turning angle close to 90° required 5 jumps to achieve. (**b**) Composite image of the initial position and four landing points in continuous forward jumping of the yellow trajectory in the line chart. Scale bar, 4 cm.

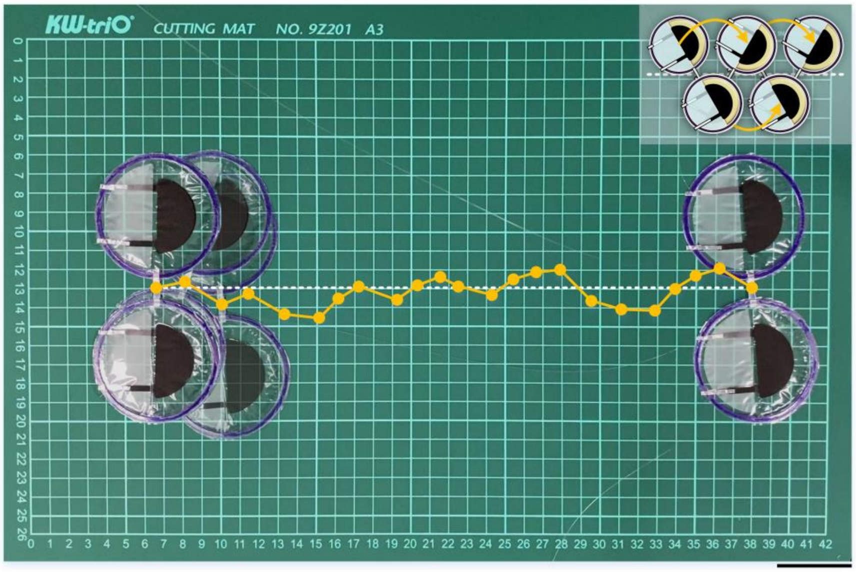

Supplementary Fig. 14. Alternate moving trajectories of the dual-body LSJR. See Supplementary Movie 8. The dual-body LSJR corrected the positions in its forward process by steering to close to the predetermined straight line at 10 kV. Scale bar, 4 cm.

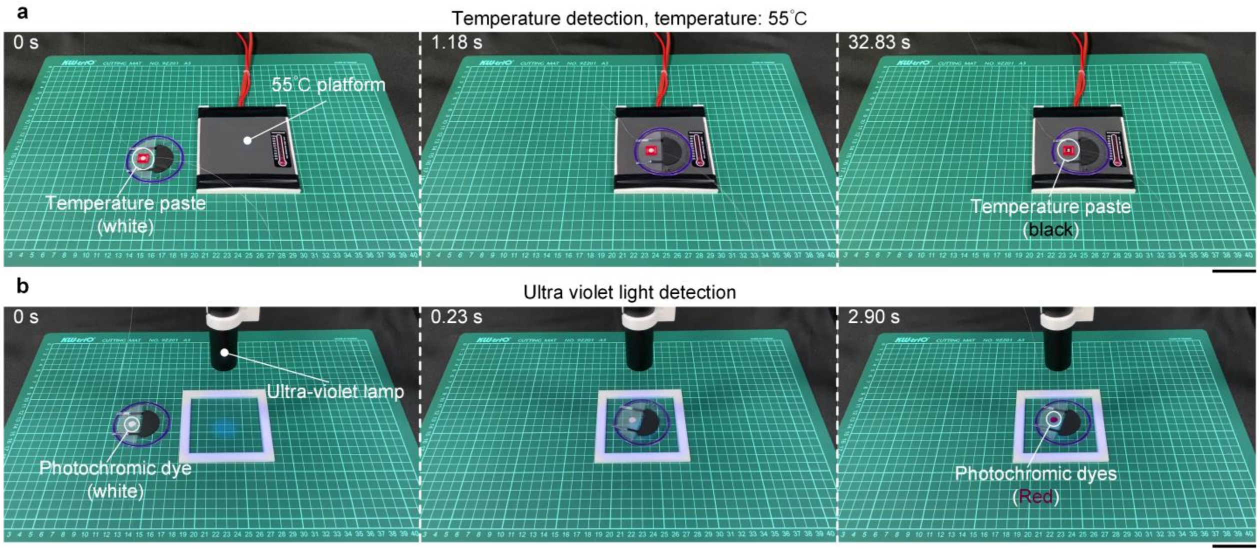

Supplementary Fig. 15. Applications of the LSJR. See Supplementary Movie 9. (**a**) Temperature detection. The color of temperature paste (40℃) changed from white to black when heated on the 55℃ platform. (**b**) Ultra-violet light detection. The color of the photochromic dye changed from white to red when exposed to ultra-violet light. Scale bar, 4 cm.
