# Supplementary material for: Legless soft robots capable of rapid, continuous, and steered jumping
Source: Nat Commun. 2021 Dec 7;12:7028. doi: 10.1038/s41467-021-27265-w (PMC8651723; doi:10.1038/s41467-021-27265-w)
Supplement: Supplementary file 3 — Description of Additional Supplementary Files [file 41467_2021_27265_MOESM3_ESM.pdf]

Description of Additional Supplementary Files

File Name: Supplementary Movie 1
Description: Jumping performance experiments of a series of designed actuators. The movie includes the single-jump experiments of three electrohydrostatic actuators without the plastic ring frame, and the influence of the frame without pre-deformation and pre-deformed frame for these actuators.

File Name: Supplementary Movie 2
Description: Characterization of a single jump. The movie includes two singlejump experiments of LSJRs without load and a load of 1 g.

File Name: Supplementary Movie 3
Description: Continuous jumping on different substrates. The movie includes the continuous jumping experiments of the LJSR at three voltages (4 kV, 7 kV and 10 kV) and three frequencies (2 Hz, 4 Hz and 8 Hz) on the PVC, and on four substrates (PVC, glass, paper and wood) at the voltage of 10 kV and 4 Hz.

File Name: Supplementary Movie 4
Description: Turning and directional jumping of the dual-body LSJR. The movie includes the turning and directional jumping experiments of the LJSR at three voltages (4 kV, 7 kV and 10 kV) of 4 Hz, and on four substrates (PVC, glass paper and wood) at the voltage of 10 kV and 4 Hz.

File Name: Supplementary Movie 5
Description: Obstacle crossing ability of the LSJR. The movie includes the crossing experiments of the single-unit LJSR for five obstacles (step, steps and gravel mound).

File Name: Supplementary Movie 6
Description: Obstacle crossing ability of the dual-body LSJR. The movie includes the crossing experiments of the dualbody LJSR for a slope obstacle by jumping forward and turning right, and a ring obstacle by adjusting direction.

File Name: Supplementary Movie 7
Description: Crossing tests for obstacles of different sizes and shapes. The movie includes the crossing tests of the single-unit LJSR for three cuboids (height of 10 mm, 14mm and 18 mm), three triangular prisms (height of 14 mm, 18mm and 22 mm) and three cylinders (height of 14 mm, 18mm and 22 mm).

File Name: Supplementary Movie 8
Description: Motion trajectories of the LSJR. The movie includes three trajectories of Continuous jumping for the single-unit LSJR, turning and alternate moving for the dual-body LSJR.

File Name: Supplementary Movie 9
Description: Applications of the LJSR. The movie includes the temperature detection with a temperature paste, and the ultra-violet light detection with a photochromic dye.
